# Artificial Intelligence-Based Hazard Detection in Robotic-Assisted Single-Incision Oncologic Surgery

**DOI:** 10.3390/cancers15133387

**Published:** 2023-06-28

**Authors:** Gabriela Rus, Iulia Andras, Calin Vaida, Nicolae Crisan, Bogdan Gherman, Corina Radu, Paul Tucan, Stefan Iakab, Nadim Al Hajjar, Doina Pisla

**Affiliations:** 1 Research Center for Industrial Robots Simulation and Testing—CESTER, Technical University of Cluj-Napoca, 400114 Cluj-Napoca, Romania; gabriela.rus@mep.utcluj.ro (G.R.); bogdan.gherman@mep.utcluj.ro (B.G.); paul.tucan@mep.utcluj.ro (P.T.); iakab.stefan@mep.utcluj.ro (S.I.); 2 Department of Urology, “Iuliu Hatieganu” University of Medicine and Pharmacy, 400012 Cluj-Napoca, Romania; iulia.andras@umfcluj.ro (I.A.); nicolae.crisan@umfcluj.ro (N.C.); nadim.alhajjar@umfcluj.ro (N.A.H.); 3 Department of Internal Medicine, “Iuliu Hatieganu” University of Medicine and Pharmacy, 400012 Cluj-Napoca, Romania; corina.radu@umfcluj.ro; 4 Department of Surgery, “Iuliu Hatieganu” University of Medicine and Pharmacy, 400012 Cluj-Napoca, Romania

**Keywords:** robotic-assisted single-incision surgery, surgical oncology, master–slave system, Hololens 2, AI agents, machine learning, YOLOv5, parallel robotic system

## Abstract

**Simple Summary:**

SILS and U-VATS procedures present many advantages, but also a series of limitations, such as the narrow operating space and poor surgeon ergonomics. To enhance the surgeon’s performance, this study aims to develop an AI and AR-based hazard detection system. The focus is on detecting and locating hemorrhages, a common occurrence especially in cancer patients. The system utilizes the YOLO v5 algorithm, trained to accurately identify bleeding in real-time. When bleeding is detected, it is displayed on a Hololens 2 device, alerting the doctor to its occurrence and origin. The system demonstrated its capability to distinguish between instances of bleeding and intraoperative irrigation, reducing the risk of false-negative and false-positive results. The current advancements in AI and AR technologies present potential of real-time hazard detection systems, which can serve as effective tools to assist surgeons, particularly in high-risk surgeries.

**Abstract:**

The problem: Single-incision surgery is a complex procedure in which any additional information automatically collected from the operating field can be of significance. While the use of robotic devices has greatly improved surgical outcomes, there are still many unresolved issues. One of the major surgical complications, with higher occurrence in cancer patients, is intraoperative hemorrhages, which if detected early, can be more efficiently controlled. Aim: This paper proposes a hazard detection system which incorporates the advantages of both Artificial Intelligence (AI) and Augmented Reality (AR) agents, capable of identifying, in real-time, intraoperative bleedings, which are subsequently displayed on a Hololens 2 device. Methods: The authors explored the different techniques for real-time processing and determined, based on a critical analysis, that YOLOv5 is one of the most promising solutions. An innovative, real-time, bleeding detection system, developed using the YOLOv5 algorithm and the Hololens 2 device, was evaluated on different surgical procedures and tested in multiple configurations to obtain the optimal prediction time and accuracy. Results: The detection system was able to identify the bleeding occurrence in multiple surgical procedures with a high rate of accuracy. Once detected, the area of interest was marked with a bounding box and displayed on the Hololens 2 device. During the tests, the system was able to differentiate between bleeding occurrence and intraoperative irrigation; thus, reducing the risk of false-negative and false-positive results. Conclusion: The current level of AI and AR technologies enables the development of real-time hazard detection systems as efficient assistance tools for surgeons, especially in high-risk interventions.

## 1. Introduction

Surgical procedures represent a major occurrence in medicine. Consequently, they are constantly evolving to keep up with the fast-paced technical advancements in this field, while also acknowledging the importance of creating efficient and optimized systems capable of meeting the high demand for medical services [1]. Among the most innovative techniques adopted to improve both the safety of the patient and the outcome of the medical procedure was the introduction of AI and AR agents in surgical procedures. 

With only one incision needed to introduce the trocars, which hold the active instruments and the laparoscopic camera, the single-incision surgery has various benefits, such as a faster recovery time, a reduced hospital stay, and superior cosmetic outcomes [2], but with a steeper learning curve. Initially reported as a procedure with higher risks in terms of surgeon ergonomics and instruments’ cross-over, the single-incision technique is now reported with similar (if not more efficient, in some cases) medical outcomes in both laparoscopic [3]—identified as SILS (single-incision laparoscopic surgery)—and thoracic surgery [4]—identified as U-VATS (uniportal video-assisted thoracic surgery). 

SILS was documented in 1969 when it was first performed in gynecology as a single incision at the umbilicus. This type of procedure only became popular in 1992, when the first report about using SILS on a considerable number of appendectomies cases was published [5]. U-VATS has also demonstrated its multiple benefits in both intraoperative and postoperative outcomes [6], showing better results compared to other approaches in lung cancer treatment [7]. 

Taking advantage of the emergence of increasingly advanced robotic systems, the surgical field quickly embraced the idea of incorporating robotics into medical procedures in order to provide surgeons with advanced tools which would: (1) increase the precision of the instruments through motion scalability, (2) increase the dexterity of the instruments by adding flexible distal joints, (3) increase the procedure ergonomics, and (4) reduce the blood loss and damage to healthy tissue. The robotic-assisted single-incision (uniport) surgery, which dates back to 1998 [8], offers many benefits, such as less trauma and a better cosmetic result, but it also exhibits some disadvantages, among which are the limited movement range of the robot platform instruments and decreased visibility. The latter are determined by the narrow space [9,10] in which the robot performs the surgery. To further improve the outcomes of SILS, a series of additional factors ought to be taken into account, such as: AI agents able to predict the optimal trajectory of the robot based on the anatomical particularity of each patient, performing image computation [11] in the pre-planning phase, and AR elements able to add further details to the surgical field.

Although the terms “Augmented Reality” and “Artificial Intelligence” were often used distinctively in surgeries, the potential to merge the capabilities of both technologies has recently sparked research interest [12]. The authors of [13] describe multiple possibilities in which Artificial Intelligence and Augmented Reality could be involved in surgical training for improved experience in young surgeons. The authors of [14] propose a surgical navigation system based on Artificial Intelligence and Augmented Reality in which the surgeon performs the medical procedure based on AI agents for image processing and surgery planning and AR agents for intraoperative navigation (based on holograms), respectively. Moreover, machine learning based on Augmented Reality systems was developed to improve surgical scene understanding and depth perception. To achieve this effect, the authors suggest a system that integrates AI and AR agents [15].

As can be seen, the integration of AI and AR agents during surgical procedures is still in its pioneering stage, with the subject presenting several prospective opportunities for the use of the above-mentioned agents in surgical procedures. A hazard identification solution for a robotic SILS technique was examined starting from the concept described in [12], especially because this topic has not been covered in the literature.

The previous studies have not focused on the integration of AR and AI in robotic SILS and U-VATS procedures, in which the demand for additional information is even higher considering the narrow space in which the surgeries are performed and the complexities associated with the master–slave structure are more numerous. Hence, the objective of this study is to address this gap by developing a comprehensive system that combines AR and AI technologies for on-site and on-time detection of intraoperative hazards. This system aims to provide surgeons with real-time information, overcoming the challenges posed by the confined surgical environment and facilitating enhanced surgical precision and decision-making. Considering that bleedings are some of the most common occurrences during surgeries, particularly for patients suffering from cancer, the study focuses on integrating bleeding identification into a real-time hazard detection system. The system utilizes machine learning algorithms capable of recognizing the bleeding and its source along with Augmented Reality and the Hololens 2 device. The objective is to alert the surgeon and to indicate the source of the bleeding by displaying it in real time. 

Following the Introduction Section above, Section 2 analyzes different types of object detection algorithms and their capabilities, followed by a comprehensive analysis of the selection of the most adequate algorithm. Furthermore, Section 3 presents the architectural structure of the system and its integration in an innovative robotic system used for SILS procedures together with the description of the software development and the YOLOv5 algorithm training. Finally, Section 4 highlights the results, while Section 5 presents the conclusions. 

## 2. Methodology

### 2.1. Critical Analysis of Hazard Detection Techniques

The hazard detection system aims to assist the surgeon by providing additional information that could be helpful, bearing in mind the complex nature of the surgical procedure. 

Considering that single-incision robotic surgery is a type of minimally invasive procedure, surgical field visualization is achieved through an endoscopic camera. The alert system is thus based on image processing. 

Image processing is extensively used in the medical field for brain tumor detection, cancer diagnosis, congenital heart defects, etc. [16]. This sector has significantly developed lately with machine learning (ML) algorithms, which now allow specialists to analyze and detect organs or anomalies faster. It is worth mentioning that the use of NN (neural network)/AI agents as fast detection tools in different areas of medicine does not imply the removal of the human expert from the medical procedure. These NN/AI agents rather represent advanced tools that can provide accurate details in a very short period of time, enabling the human expert to make better medical decisions. 

Therefore, this paper adopted an approach in which object detection methods were used to detect bleeding in real time. The goal of the object detection algorithms is to locate the target and to return a bounding box that shows the item’s location. These algorithms are used for a variety of tasks, in which the goal is based on event detection, target tracking, semantic analysis, or scene interpretation [17]. 

There are multiple methods used to perform object detection, and in the last decade, due to the advance of ML, these algorithms have become faster and faster, performing the task with a high degree of accuracy. 

#### 2.1.1. SSD (Single-Shot Detector)

The SSD algorithm is based on a feed-forward convolutional network able to generate a collection of bounding boxes around the detected object, scoring the possibility that the detected object belongs to a certain class [18]. The structure of the model is made up of two parts: the backbone model based on a pre-trained image classification network, from which the final classification layer is removed, and the SSD head, attached to the top of the backbone model, being made up of convolutional layers. The model takes the input image and divides it into grids, thus performing detection for each grid. The process is followed by the application of a non-maximum suppression model to achieve a final detection from the set of overlapping detections.

#### 2.1.2. Faster R-CNN (Faster Region-Based Convolutional Network)

The Faster R-CNN model is a complex algorithm composed of three neural networks (NN): the feature network, region proposal network (RPN), and the detection network. The input image is processed by the feature network (a pre-trained image classification network), which aims to retrieve the features from the image. At the same time, RPN (a simple NN with convolutional layers) generates a number of bounding boxes, named regions of interest (ROI). These two NNs serve as input for the detection network, which takes the input from both networks and generates the final class and bounding box [19].

#### 2.1.3. YOLOv5

The YOLO (You Only Look Once) algorithm, released for the first time in 2015, represents a viable solution for object detection [20]. The originality of YOLO lies in the mechanism by which the detection is performed, using a single fully connected layer to create predictions of bounding boxes and class probabilities all at once. This differs from earlier systems that repurposed classifiers to perform detection [21]. In order to improve the accuracy and the speed of the algorithm, many versions of YOLO have been released over the years [22]. 

**Figure 1:**
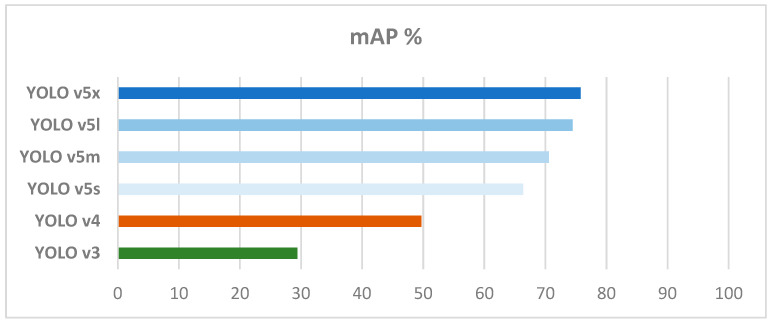
Performance of YOLOv3, YOLOv4, and the four versions of the YOLOv5 algorithm, based on accuracy, based on the content from Figure 1 in [24].

YOLOv5, released in 2020, presents a complex structure based on the EfficientNet network architecture. The performance of this approach has been enhanced by the inclusion of two key features: spatial pyramid pooling (SPP), which has improved the identification of small objects, and dynamic anchor boxes, which allowed for better alignment of the bounding boxes with the identified object [23]. The efficiency of YOLOv5 on different tasks was extensively studied [24,25], with the results showing that this version of YOLO presents satisfactory accuracy and, more importantly, a superior speed in detection, as seen in Figure 1. 

**Figure 2:**
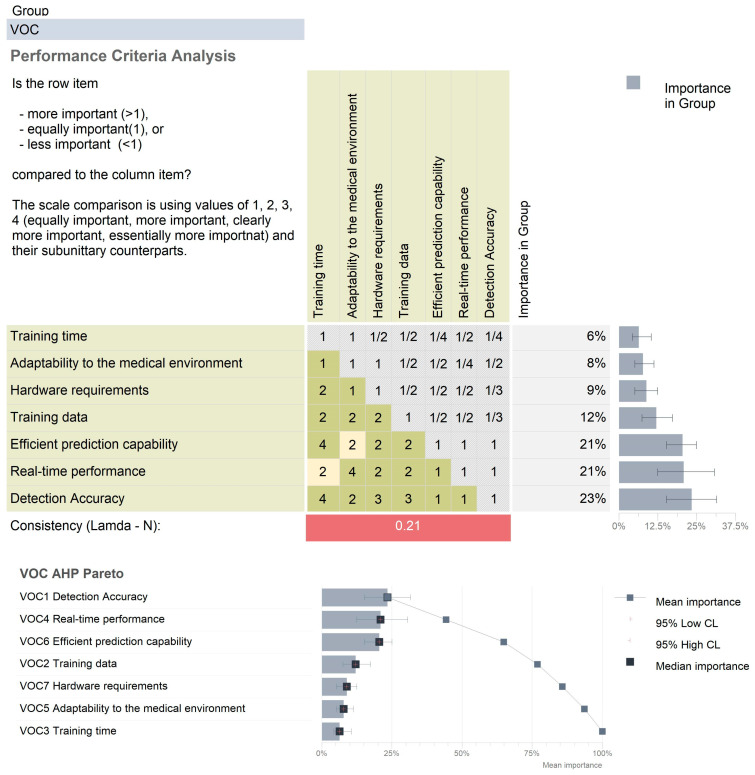
The AHP analysis on the critical performance criteria of AI algorithms.

Furthermore, an in-depth analysis meant to enable the proper selection of the most efficient AI algorithm to train the neural network for hazard detection was proposed. In order to achieve this, the authors have identified, with the support of medical experts, a set of performance criteria relevant to the task at hand. These performance criteria were then evaluated in terms of criticality levels using the analytic hierarchy process (AHP), presented in Figure 2. This methodology was subsequently followed by a Pugh selection methodology (using the Qualica^®^ software) that will assess the three methods described in the paper, with the purpose of identifying the most efficient approach. 

The identified performance criteria are presented below, followed by a brief motivation for their selection:

Detection accuracy. Accuracy detection is critical for the safety of the patient when targeting specific hazards during the surgical intervention (in this case, internal bleeding). While a false-positive result (a false identification of bleeding) may be disturbing for the surgeon, a false-negative one is unacceptable, as timely identification of the bleeding can save the patient’s life. 

Training data. Each AI algorithm uses training datasets to learn, and in the case of internal intraoperative bleeding, the data are difficult to collect, being mostly retrieved from live recordings of procedures in which incidents occur. As this information comes from multiple sources, there is uncontrolled variability in the dataset. This requires the AI algorithm to be robust to these variations. 

Training time. The training time of a neural network depends on many parameters derived from the input data (which are assumedly similar), the network configuration, and the computer hardware. While the training time is not relevant for a final implementation, it becomes important during the study and research phase, as the neural network needs to enable multiple tests, configurations, etc. Therefore, the final goal of the training time is accuracy maximization.

Real-time performance. The hazard detection system is used during the surgical procedure in a dynamic field of view. The most dangerous bleeding is the one that occurs at the extremities of the surgical field (since in the central part where the surgeon is actively working, it will be easily noticed). Such bleeding is sometimes displayed for only a few seconds. Thus, bleeding detection should occur as soon as possible, with a targeted time delay of less than 0.1 s. 

Adaptability to the medical environment. One parameter which differentiates AI algorithms refers to their capabilities to either perform “object detection” or perform “image classification”, with the latter proving to be more effective in medical applications.

Efficient prediction capability. As mentioned before, even though bleeding is marginally the same, its illustration can vary a lot depending on the location, type of vessel, neighboring organs, camera type, lighting, overshadowing, etc. Ideally, the hazard detection tool should be invariant to these changes. Thus, it is important to minimize the false results or to very accurately identify the limitations of each algorithm. 

Hardware requirements. An important aspect in the development of complex control platforms is the efficient use of hardware resources to manage all functionalities in real time. Thus, a lower CPU/GPU/RAM consumption is targeted as this hazard detection tool must run in real time during the entire procedure. 

Additionally, a Pugh Matrix was used to evaluate the three potential candidates for the development of the AI hazard detection tool: SSD, Faster R-CNN, and YOLOv5. 

Using the scale in the upper left corner, each of the three algorithms were assessed with respect to the weighted values of the performance criteria. 

**Figure 3:**
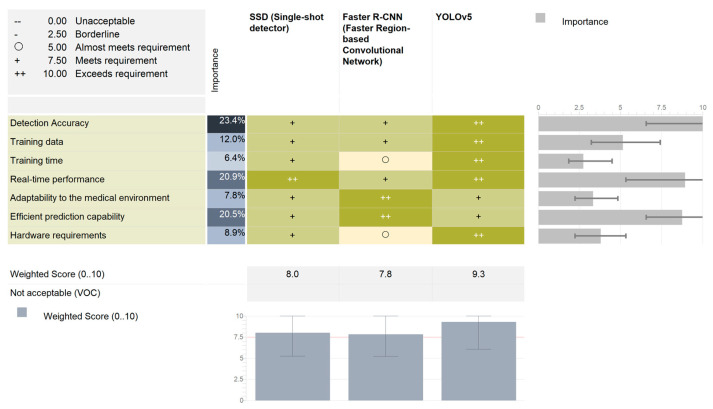
The Pugh Matrix results for the weighted analysis of the three candidate algorithms.

As seen in Figure 3, while all three algorithms met the pre-defined requirements, YOLOv5 has the potential to deliver the best possible results. SSD exhibited a very good real-time performance. However, it lacks accuracy and has higher hardware requirements. The most accurate algorithm was the Faster R-CNN, which uses a two-stage image analysis. However, this analysis rendered it less suitable for real-time applications. 

### 2.2. Detection System Architecture

**Figure 4:**
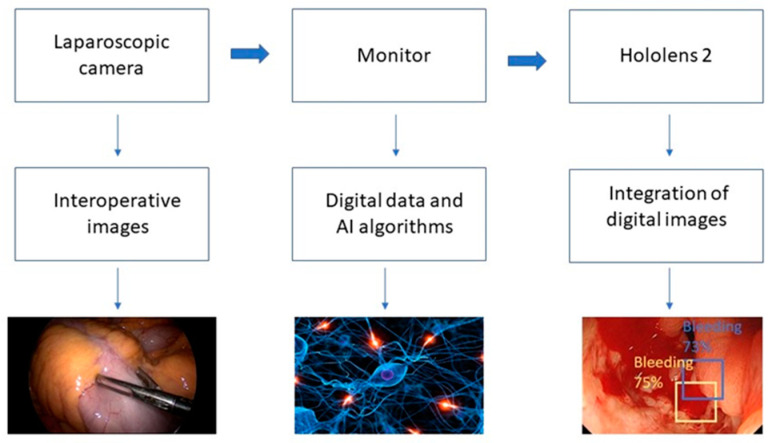
The schematic representation of the hardware system composed of a laparoscopic camera, monitor, and the Hololens 2 device.

The hardware system workflow proposed for bleeding/hemorrhage detection is presented in Figure 4. The endoscopic camera located in the surgical field retrieves images and sends them to the connected PC, where they are displayed on a monitor. Using the algorithm, the surgeon, wearing the Hololens 2 device, is presented with the bleeding and its source through the bounding boxes, created as holograms on the device. 

This system is designed to fit with the robotic master–slave concept, in which the main surgeon sits at a master console controlling the surgical instruments by actuating the slave robotic system. 

The Hololens 2 device was released in 2019 by Microsoft [26] and uses Augmented Reality technology to overlap holograms into the real word. This device presents many advantages, such as high autonomy, eye tracking, and its own operating system, thus supporting the development of advanced applications in many fields. In robotic-assisted surgery, Hololens 2 was used to assist in surgical navigation based on holographic markers [27] to explore the possibility of replacing real monitors with virtual ones, on which images from the endoscopic camera are displayed [28]. Another application of Hololens 2 was to create simulators through which the surgical robots can be tested in safe conditions in the prototype phase [29].

This AI-based hazard detection tool is one of the intelligent components embedded in the master console of a new robotic system for SILS currently under development. A brief description of the slave component is presented below.

#### PARA-SILS-ROB

**Figure 5:**
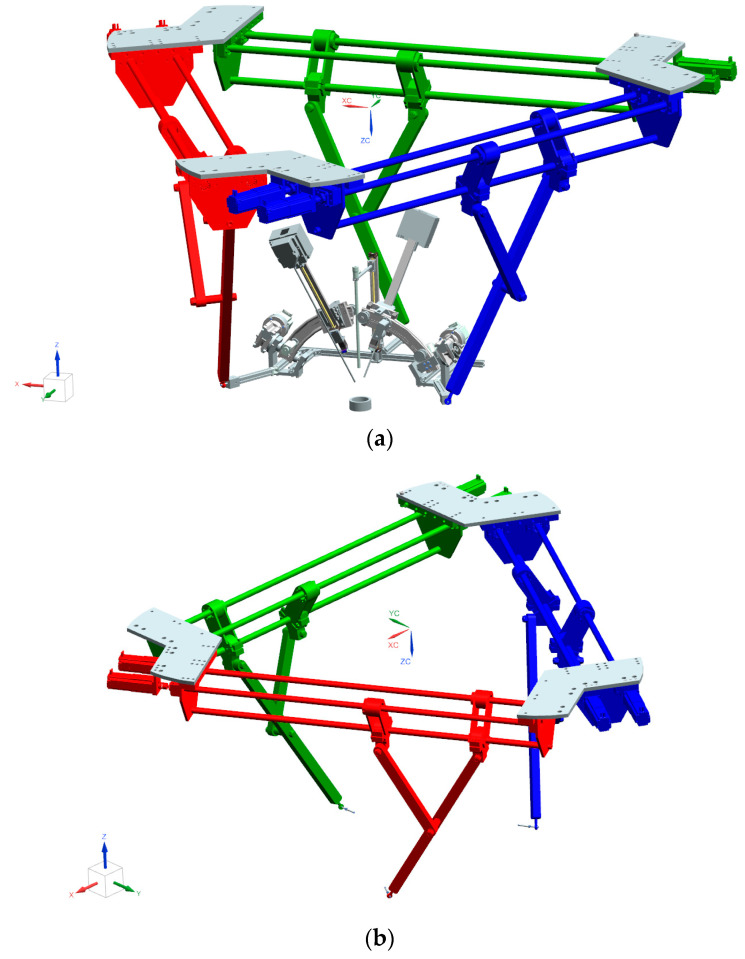

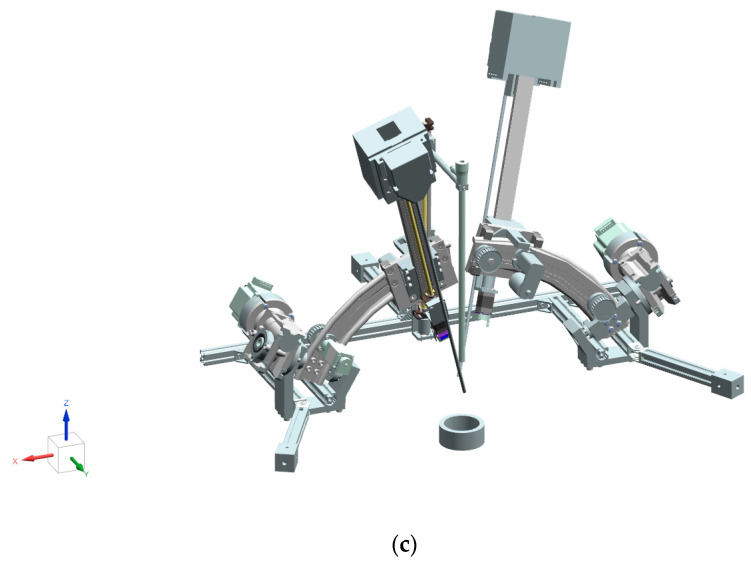
(**a**) Design of the PARA-SILS-ROB robotic system. (**b**) The 6-DOF parallel robot. (**c**) Mobile platform with 3-DOF.

An innovative modular robotic system (Figure 5a) for SILS was proposed by the authors of [30]. It consists of several modules: a 6-DOF (degrees of freedom) parallel structure, presented in Figure 5b, which manipulates a mobile platform (Figure 5c), on which a 1-DOF module located in the center of the platform is used for endoscopic camera insertion, and two 3-DOF modules are located on the sides of the central one, which achieve the orientation and insertion of the active surgical instruments. 

Summarizing the detailed description of the robotic system in [30,31,32], the motions of the robot can be described as follows:

(a) Motions outside the body: The 6-DOF parallel robot positions and orients the mobile platform carrying the instruments, aiming to move the tips of the three instruments over the insertion points in the body (using any model of a SILS port). The active instruments’ modules will orient the active instruments’ tips to ensure that the distance between their tips and the tip of the endoscopic camera is consistent with the SILS port. This motion is achieved without any restrictions.
(b) Instruments’ insertion into the body: The instruments’ modules will insert the instruments based on predefined depths on linear trajectories. In this step, restrictions are applied onto the robotic system, as follows: (1) The 6-DOF parallel robot will register the insertion point coordinates of the endoscopic camera, which will be used as a fixed point in the robot workspace from this moment on until the instruments will be removed from the body—based on the principle of remote center of motion (RCM) [33,34]. (2) The 6-DOF parallel robot will preserve its vertical axis coordinates. The instruments’ insertion inside the body is performed only by the insertion modules.
(c) Motions inside the body: The 6-DOF parallel robot will achieve the orientation of the endoscopic camera (preserving the fixed point, defined earlier as RCM). The orientation of the active instruments is achieved through the orientation function of the active instruments’ modules, which achieve the RCM through kinematic constraints.

**Figure 6:**
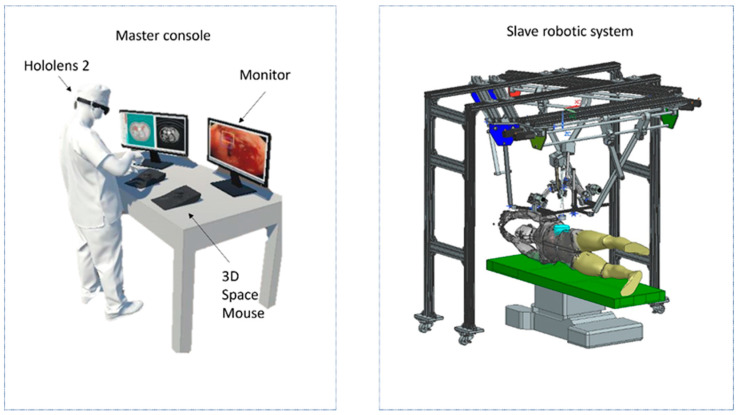
Integration of a hazard detection system in the SILS robotic platform, including the master–slave concept.

A schematic representation of the integration of the Hololens device in the master–slave system is presented in Figure 6. The surgeon is comfortably positioned at the master console and is aided by two 3D Connection Space-Mouse devices to control the active instruments of the robotic system, and the camera position can be controlled either by voice commands [35] or by using one of the 3D devices. Benefiting also from the integration of the Hololens 2 AR device, the surgeon can receive, superposed over the intraoperative images, different hazards detected in real time by the AI algorithms, such as the source of the bleeding and blood loss estimation (scaled based on potential risks).

### 2.3. Software Development and Testing

#### 2.3.1. Dataset

The quantity and quality of the training data have a direct impact on the robustness of an ML algorithm, affecting both the accuracy of the prediction and the bias-proneness of the algorithm.

A total of 15 videos were selected for this study, containing different laparoscopic surgeries in which bleeding was present. The selection of videos was based on three major factors:

(a) The video had to contain the onset of bleeding. In this way, the algorithm was trained to detect the bleeding from the start and to mark its location.
(b) It had to contain both scenes where the bleeding was present and where it was not. The images where the bleeding was not present were used as negative samples to avoid the overfitting of the model and to increase the accuracy.
(c) The clip needed to offer the possibility to track the event—the bleeding event had to be visible (without many juxtapositions) to create (if needed) a trace of the bleeding.

Using the OpenLabelling tool [36], the images were retrieved from the selected videos at 20 frames per second, thus obtaining a total of 2694 images, out of which 1353 contained bleeding events. The images were in a 640 × 640 format, this being specific for the YOLOv5 (YOLOv5m and YOLOv5s) input tensor. The same tool was also utilized to annotate the bleeding. For the purpose of this paper, a single class named “bleeding” was used, and the data resulting from the annotation process (the coordinates of bounding boxes) were saved in YOLO Darknet annotation format (four values: coordinates for X- center, Y-center, and dimensions for width and height). 

The annotation was assisted by a person specialized in providing valid data. 

#### 2.3.2. Augmentation 

**Figure 7:**
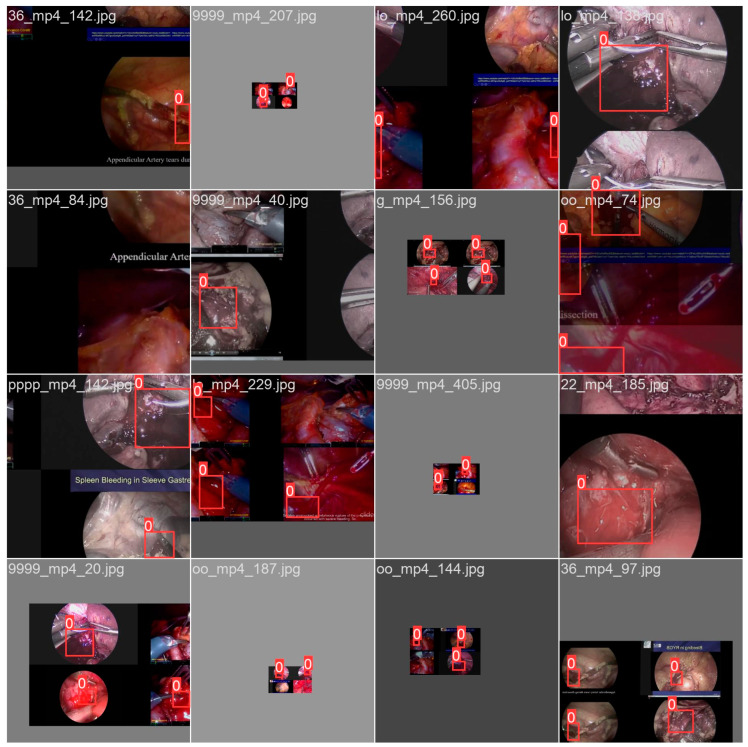
Results of augmentation operations, including scaling, rotation, cropping, color augmentation, and mosaic augmentation.

In order to avoid overfitting and to increase the performance of the algorithm, a set of additional operations was required to augment the input data. For this dataset, augmentation techniques were used, such as scaling to obtain features with similar value ranges and to prevent bias, cropping to obtain large input data focused on the bleeding events, and rotating to create opportunities for the algorithm to detect bleeding regardless of the context and the position in which they occur. Color augmentation (Gamma correction and brightness) was used to achieve different contrast levels, thus increasing the adaptability of the algorithm to different types of laparoscopic cameras, and mosaic augmentation was used to collect inputs in which the bleeding event was obstructed (since the mosaic technique combines different training inputs in a single image in which the object, or in this case the bleeding, could be overlapped) and to offer images where bleeding was present in different backgrounds and contexts, as seen in Figure 7. 

The total dataset used in the training process was split into three parts: 270 images were used for testing, 2155 were used for training, and 269 were used for validation.

Training 

In order to obtain a loss function (difference between the prediction of bounding boxes and the ground truth) as low as possible, the model was trained in different configurations, in which the hyperparameters were adjusted, as seen in Table 1. 

The custom data were trained using both the YOLOv5s and YOLOv5m models, as these configurations of the YOLOv5 model are the most suitable for the purpose of this study, considering the processing time and the hardware resources used in medical applications. Both configurations require images with the size of 640 × 640 pixels, with the major difference between them being their size and complexity. YOLOv5s is smaller and faster but less performant from the perspective of accuracy compared to YOLOv5m, which exhibits a robust architecture and a high level of accuracy, but the major disadvantage being the increased time used for computation and training. 

The hyperparameters were changed considering the performance of the training process, as follows: The first training session was conducted with a learning rate of 0.1, a batch size of 8, and the total number of epochs was set to 100. In the following sessions, only one parameter was changed, while the rest of the values remained unaltered (e.g., Session 2: learning rate 0.05, batch size 8, epochs 100), thus obtaining a clear idea of the best configuration of parameters for the obtention of the best model accuracy. The configurations were tested with the two versions of the YOLOv5 model and with both SGDM and Adam optimizers. 

The training sessions were performed on a NVIDIA RTX A2000 GPU utilizing Python 3.11 and the Pytorch library (2.0.1 + cu117).

#### 2.3.3. TCP/IP Connection 

**Figure 8:**
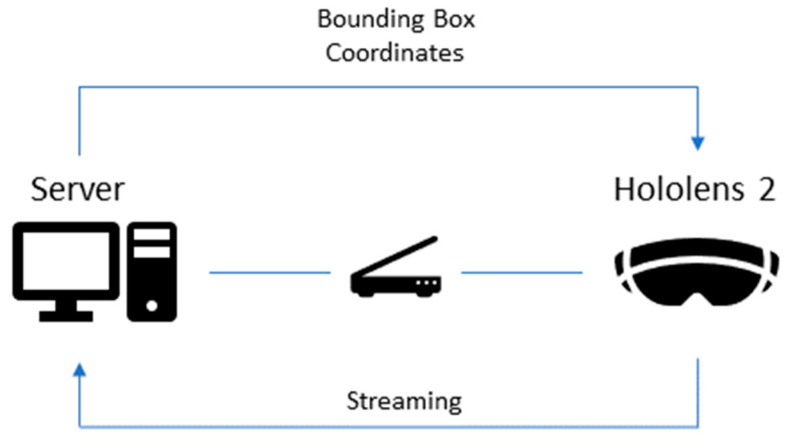
Schematic representation of TCP/IP communication, including the server located on the PC, the Hololens device, and the communication between them based on TCP/IP communication.

Due to the reduced computational resources of the Hololens 2 device, the system was established on the TCP/IP (Transmission Control Protocol/Internet Protocol) communication protocol presented in Figure 8. This was prompted by the fact that several trained networks were tested by direct implementation on the Hololens 2 device, but none had satisfactory results in terms of real-time detection, which imposed the transfer of the inference process on the computer (running as a server). 

Taking into account the high resource requirements for real-time detection, the server was setup on a PC (CPU Intel^®^ Core™ i9-12900KS and Nvidia RTX A2000 GPU) and the client socket was established on Hololens. The workflow, therefore, was as follows: Python received frames as input from Hololens through streaming, with these being processed by YOLOv5m. After the detection process for a frame is completed, the output (bounding box coordinates) is sent to the Hololens, which displays them in the form of boxes.

In addition to Python, the Unity Engine platform was also used. Considering that the Unity platform offers adequate support for AR applications, this environment was chosen to create the application, which incorporates both the client socket and the implementation of the drawing of the bounding box process. Furthermore, it also calibrates the boxes with the environment of the user and takes into account factors such as the offset between the camera of Hololens and the eyes of the user, the understanding of 3D space, and the box size. 

## 3. Results

The main purpose of this study was to create a real-time hazard detection system using AI algorithms meant to increase the safety of the patient and the efficiency of the surgical procedure. As a demonstration, the authors selected the intra-operative hemorrhage which, if left uncontrolled, can have drastic consequences. The YOLOv5 algorithm has been identified as the best option with respect to the performance criteria for this specific task. Furthermore, in order to illustrate the cross-platform capabilities of the solution, the Hololens 2 device was selected as the AR device.

Once the training period was over, the results retrieved for each individual configuration were analyzed in order to determine the optimal configuration for this type of task. The configuration which achieved the highest level of accuracy is highlighted in Table 1 (blue color). 

**Figure 9:**
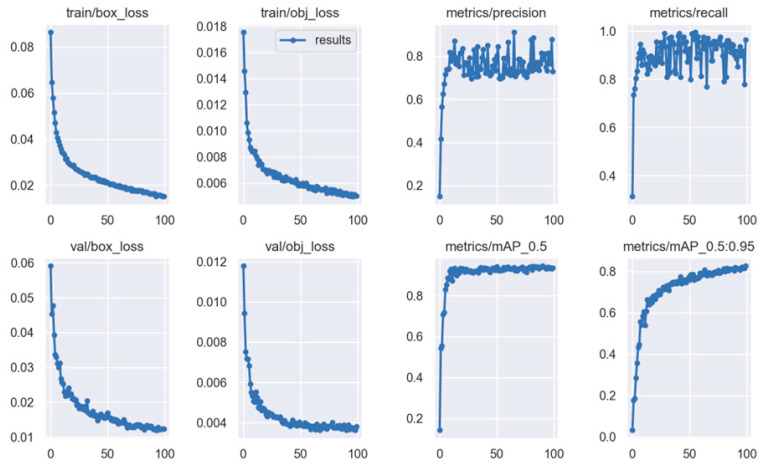
Detection performance of YOLOv5m using the box-loss value associated with bounding box predictions, obj_loss based on the predicted loss score, and the ground truth label, precision, recall, and mean average precision (thresholds of IoU starting from 0.5 and increasing to 0.95).

As seen in Figure 9, with the configuration presented above, the detection performance of the model achieved the values summarized in Table 2.

**Figure 10:**
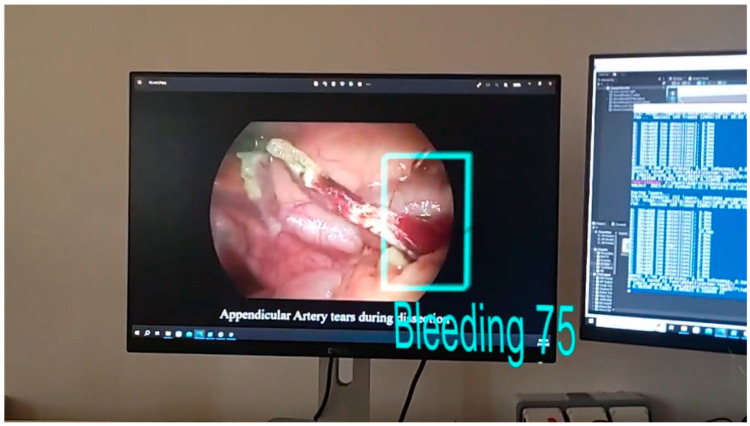
Bleeding detection (debut phase) displayed on the Hololens 2 device. The confidence of the detection was 75%.

**Figure 11:**
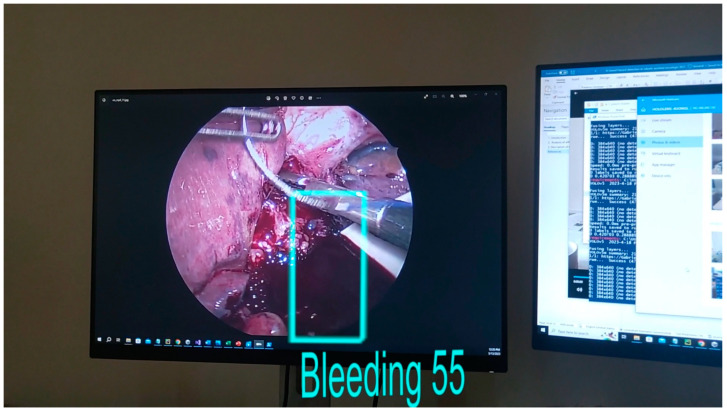
Bleeding detection displayed on the Hololens 2 device.

The model was tested using the IoU (Intersection of Union) method with a threshold value set to 0.45. Figure 10 and Figure 11 present the bounding boxes and the confidence of the detection seen through Hololens. It can be noticed that the algorithm is capable of detecting the bleeding both in its incipient phase and as it progresses (if it is not stopped).

The average detection time was below one second which, for this type of event, can be considered real time. 

## 4. Discussion

Robotic-assisted single-incision surgery is a complex procedure that demands innovative approaches to enhance its efficiency and to ensure patient safety. This requires the use of new technologies to optimize the surgical intervention.

With the goal of improving efficiency, advancements in robotic technology enable precise and controlled movements during the surgical procedure. By utilizing robotic structures with enhanced dexterity and accuracy, surgeons can perform intricate maneuvers with greater ease. These robotic systems often incorporate real-time imaging and feedback mechanisms, providing surgeons with a comprehensive view of the surgical site and enhancing their ability to make informed decisions. 

The continuous advancements in technology and medicine have paved the way for synergistic outcomes in surgical interventions. Considering the substantial risk of bleeding, especially in oncological surgery, it becomes imperative to create a system in which bleeding can be quickly detected, contained, and stopped. 

While intraoperative bleeding (hemorrhages) can be a major problem in most surgical interventions, it has become even more critical in oncologic surgery due to the higher incidence. Cancer patients are more exposed to this risk based on multiple causes:

- Neo-adjuvant treatments. Very often, cancer patients undergo chemotherapy and/or radiation therapy [37], targeting tumor shrinkage (turning an inoperable tumor into a manageable one), which has uncontrollable side effects on the neighboring tissues.
- Tumor location. As tumors have higher vascularity or location in the proximity of large blood vessels, the surgical intervention may often cause unwanted bleeding, which is more prone to uncontrollable hemorrhages [38].
- Blood-thinning medication. As shown in [39], cancer patients have a 50% to 70% higher risk of bleeding compared to non-cancer patients (with the same anticoagulant).

By harnessing the power of the latest technologies, such as AI and AR, the development of such a system becomes feasible, offering real-time detection and providing surgeons with critical information to effectively manage bleeding complications.

The system presented in this paper aimed to address multiple issues in surgery: (1) to prove the real-time capabilities and efficiency of AI/AR systems, (2) to assess the quantity of information supplied to the surgeon, (3) to assess the level of “intrusion” of the system into the surgical intervention, (4) to evaluate the interaction between the surgeon and an interactive digital system throughout the surgery, and (5) to emphasize the potential role of AI/AR systems as hazard detectors in the improvement of patient safety and surgical outcomes.

The findings of this study demonstrated the successful utilization of the YOLOv5 algorithm for blood detection, offering key features such as speed, accuracy, and tracking capabilities. These characteristics play a crucial role in effectively addressing the requirements of blood detection in surgical procedures.

The YOLOv5 algorithm proved to be highly efficient in swiftly identifying bleedings in real time, enabling timely interventions in critical situations. Its accuracy in detecting hemorrhages ensures reliable results, thus reducing the risk of false negatives or false positives. Additionally, the algorithm’s tracking capability allows for continuous monitoring of the bleeding, facilitating the assessment of its progression and enabling appropriate intervention strategies.

The speed of the YOLOv5 algorithm is of utmost importance in surgical settings, where immediate action is often necessary to mitigate bleeding complications. Its ability to quickly process and analyze video data streamlines the detection process, providing prompt alerts to surgeons and enabling them to take timely measures to address the bleeding.

Incorporating the Hololens 2 device as a valuable component of the system further enhanced its capabilities alongside the Artificial Intelligence algorithms. This device offers a distinct advantage by providing augmented information that supplements the surgeon’s natural field of view through image superposition. By seamlessly overlaying digital information onto the surgeon’s visual perception, the Hololens 2 device enriches the surgical experience, offering real-time insights, anatomical guidance, and critical data that may not be readily visible to the naked eye. 

A possible scenario involves several Hololens 2 devices being connected to the same server, enabling several members of the medical team to have simultaneous access to the information. In this way, the surgical team can decide who is viewing this additional information (hazard detection) without necessarily displaying it on the main surgeon’s console, allowing the assistant surgeon to decide whether to warn the principal surgeon on the identified hazard or not.

While Hololens 2 offers numerous advantages for the medical field, it is crucial to critically assess its limitations, particularly its constrained hardware capacity, which poses challenges for developing standalone applications. One notable drawback is the difficulty in creating self-contained applications. For instance, a bleeding detection system was tested on the Hololens 2 device as a standalone application, thus eliminating the need for a server. Various approaches were explored to implement the algorithm on the device, including multi-threading, frame processing, and utilizing a smaller model. However, despite these attempts, the time required for detection using the installed application was significantly prolonged, rendering it impractical for real-time detection.

The system can be improved in the future by incorporating functions to detect other major or unwanted events that may occur during surgery; for example, analyzing the quality of sutures or monitoring small surgical objects (e.g., clips) dropped inside the patient, while also indicating the location where they have fallen (although these cases are very rare, they can have critical consequences).

With an emphasis on high-risk procedures such as curative or palliative tumor resections, future research and improvement of this tool can prove beneficial to all forms of robotic-assisted surgery and even in manual, minimally invasive surgery, with excellent outcomes for all patients.

## 5. Conclusions

The continuous advancements in technology and medicine have enabled the achievement of synergies with real positive outcomes for surgical interventions. Considering both the complexity of SILS procedures, performed with a master–slave structure as the one presented in this paper, and the frequency of hemorrhages in these types of procedures, especially in people suffering from cancer, the authors proposed the use of AI algorithms integrated into an AR device (Hololens 2) to serve as a real-time detector of intraoperative hazards. By analyzing multiple algorithms, the authors have identified YOLOv5 as a good candidate for real-time detection of abnormalities, displaying a very good accuracy, a sufficient prediction efficiency, and very good response times, with all the above having been tested in the detection of intraoperative bleeding. 

The system underwent testing for both early-stage bleeding and advanced bleeding scenarios. The results indicated that the detection times and accuracy were deemed optimal, enabling the system to be effectively utilized for real-time bleeding detection, encouraging, at the same time, its further development.

## Figures and Tables

**Table 1:** Parameters considered for optimization of the YOLOv5 algorithm.

Model	Learning Rate	Batch Size	Optimizer	Epochs
YOLOv5s **YOLOv5m**	0.1 0.05 **0.001**	**8** 16 32	**SGDM** Adam	100 **250** 350 500

**Table 2:** Detection performance of YOLOv5m.

Train/ Box_Loss	Train/ Obj_Loss	Metrics/ Precision	Metrics/ Recall	Metrics/ mAP_0.5	Metrics/ mAP_0.5:0.95	Val/ Box_Loss	Val/ Obj_Loss
0.015182	0.0049918	0.7293	0.9641	0.93654	0.82633	0.012306	0.0038008

## Data Availability

Data sharing is not applicable to this article.
